# Genetic substructure and admixture as important factors in linkage disequilibrium‐based estimation of effective number of breeders in recovering wildlife populations

**DOI:** 10.1002/ece3.3577

**Published:** 2017-11-07

**Authors:** Alexander Kopatz, Hans Geir Eiken, Julia Schregel, Jouni Aspi, Ilpo Kojola, Snorre B. Hagen

**Affiliations:** ^1^ NIBIO—Norwegian Institute of Bioeconomy Research Svanvik Norway; ^2^ Department of Biology University of Oulu Oulu Finland; ^3^ Natural Resources Institute Finland (Luke) Rovaniemi Finland

**Keywords:** brown bear, effective population size, temporal genetic analysis, *Ursus arctos*

## Abstract

The number of effective breeders (*N*_*b*_) and effective population size (*N*_*e*_) are population parameters reflective of evolutionary potential, susceptibility to stochasticity, and viability. We have estimated these parameters using the linkage disequilibrium‐based approach with LDNE through the latest phase of population recovery of the brown bears (*Ursus arctos*) in Finland (1993–2010; *N *= 621). This phase of the recovery was recently documented to be associated with major changes in genetic composition. In particular, differentiation between the northern and the southern genetic cluster declined rapidly within 1.5 generations. Based on this, we have studied effects of the changing genetic structure on *N*_*b*_ and *N*_*e*_, by comparing estimates for whole Finland with the estimates for the two genetic clusters. We expected a potentially strong relationship between estimate sizes and genetic differentiation, which should disappear as the population recovers and clusters merge. Consistent with this, our estimates for whole Finland were lower than the sum of the estimates of the two genetic clusters and both approaches produced similar estimates in the end. Notably, we also found that admixed genotypes strongly increased the estimates. In all analyses, our estimates for *N*_*e*_ were larger than *N*_*b*_ and likely reflective for brown bears of the larger region of Finland and northwestern Russia. Conclusively, we find that neglecting genetic substructure may lead to a massive underestimation of *N*_*b*_ and *N*_*e*_. Our results also suggest the need for further empirical analysis focusing on individuals with admixed genotypes and their potential high influence on *N*_*b*_ and *N*_*e*_.

## 1 INTRODUCTION

The effective population size (*N*_*e*_) is reflective of a population's evolutionary potential, its susceptibility to stochastic processes, and therefore survival. While the census size of a population (*N*_*c*_) is an estimate of the population size, *N*_*e*_ is an estimate of the number of individuals contributing offspring to the next generation (Charlesworth, 2009). Assessing *N*_*e*_ is complex, and estimation from demographic data is ambiguous without data on individual reproductive success (Leberg, 2005). However, genetic information enables direct estimation of *N*_*e*_ (Harris & Allendorf, 1989; Luikart, Ryman, Tallmon, Schwartz, & Allendorf, 2010; Palstra & Fraser, 2012), which is defined as the size of an idealized population which causes the same magnitude in random genetic drift as the population in question (Fisher, 1930; Wright, 1931). From this definition, three genetic approaches to estimate *N*_*e*_ have been described: inbreeding *N*_*e*_, variance *N*_*e*_ (Crow & Denniston, 1988), and eigenvalue *N*_*e*_ (Ewens, 1982). These approaches differ theoretically and may deliver different results, for example, if the population is not temporally stable (Ewens, 1982; Luikart et al., 2010; Orrive, 1993; Wang, 2005). Additionally, there is coalescent *N*_*e*_ (Sjödin, Kaj, Krone, Lascoux, & Nordborg, 2005), which is based on neutral genetic theory and showed to work soundly for small populations (for review see, e.g., Berthier, Beaumont, Cornuet, & Luikart, 2002; Anderson, 2005; Luikart et al., 2010; Hare et al., 2011; Kimberly & Whitlock, 2015; Wang, Santiago, & Caballero, 2016).

Changes of *N*_*e*_ over time have been traditionally estimated using two‐sample *N*_*e*_‐estimators as the pseudomaximum likelihood method (MLNE, Wang, 2001), temporal *F*‐statistics (*N*_*e*_‐estimator, Do et al., 2014 or TempoFs, Jorde & Ryman, 2007), or the coalescent Bayesian temporal method (TM3, Berthier et al., 2002; Co *N*_*e*_ Anderson, 2005). All these methods analyze allele frequency changes caused by genetic drift between two different points in time, preferentially several generations apart (Leberg, 2005; Luikart et al., 2010). As of late, single‐ or one‐sample methods, based, for example, on linkage disequilibrium (LDNE, Waples & Do, 2008), approximate Bayesian computing (O *N*_*e*_ SAMP, Tallmon, Koyuk, Luikart, & Beaumont, 2008), parentage (AgeStruct, Wang, Brekke, Huchard, Knapp, & Cowlishaw, 2010), or sibship assignment (Colony2, Wang, 2009) have been applied for temporal tracking of *N*_*e*_ based on annual samples of genotypes (see, e.g., Baalsrud et al., 2014; Jansson, Ruokonen, Kojola, & Aspi, 2012; Kamath et al., 2015; Schregel et al., 2012; Skrbinšek et al., 2012). If temporal sampling over a time gap of several generations is not possible, single‐sample methods may be more precise in estimating *N*_*e*_ (Wang et al., 2016; Waples & Do, 2010). Like two‐sample estimators (Schwartz, Luikart, & Waples, 2007; Waples & Yokota, 2007), single‐sample estimators assume discrete generations (Waples, Antao, & Luikart, 2014). Thus, for species with overlapping generations, the effective number of breeders (*N*_*b*_) is often easier to estimate. In this case, *N*_*b*_ rather reflects the number of individuals of one breeding season or reproductive cycle which produced the analyzed cohort of offspring (Waples, 2005; Waples & Antao, 2014). Both parameters are related, and *N*_*e*_ can be estimated using *N*_*b*_ as proxy, but that relationship is complex (Waples, Luikart, Faulkner, & Tallmon, 2013; Waples et al., 2014). Recently, it has been shown that life‐history traits are crucial factors influencing *N*_*b*_ and *N*_*e*_ as about half of the variance in *N*_*b*_ and *N*_*e*_ can be explained by two life‐history traits: age at maturity and adult life span (Waples et al., 2013, 2014). *N*_*b*_ and *N*_*e*_ can be corrected for bias quantitatively with information on these two traits (Ruzzante et al., 2016; Waples et al., 2014). *N*_*b*_ is representative of *N*_*e*_ for one reproductive season (Duong, Scribner, Forsythe, Crossman, & Baker, 2013; Waples, 2005; Waples & Antao, 2014), and *N*_*b*_ and *N*_*e*_ are both influenced by the same population dynamics, although temporal scales may vary: Where *N*_*e*_ reflects long‐term evolutionary processes, *N*_*b*_ indicates more short‐term eco‐evolutionary processes (Waples, 2002; Waples et al., 2014).

The linkage disequilibrium (LD)‐based method LDNE (Waples & Do, 2008) is a robust single‐sample estimator of *N*_*b*_ and *N*_*e*_ frequently applied in conservation genetic studies (Gilbert & Whitlock, 2015; Palstra & Ruzzante, 2011; Wang et al., 2016; Waples & Do, 2010). The extent of LD in a population, that is, the nonrandom distribution of alleles over different loci, is influenced by fragmentation, bottleneck events, and migration and therefore affects the estimation (Antao, Pérez‐Figueroa, & Luikart, 2011; England, Luikart, & Waples, 2010; Slate & Pemberton, 2007; Slatkin, 1994; Waples & England, 2011). In nature, population subdivision often results in genetic drift by nonrandom mating of individuals, while migration can lead to homogenization among subpopulations. Simulation studies have demonstrated that for Wright's island model, the global *N*_*e*_ (or meta‐*N*_*e*_; Fraser et al., 2007) may increase above the sum of local or deme *N*_*e*_ of the subpopulations (∑*N*_*e*(s)_), while asymmetrical migration may have the opposite effect (Whitlock & Barton, 1997; Wang & Caballero, 1999; Tufto & Hindar, 2003; Fraser et al., 2007; Palstra & Ruzzante, 2011; Hare et al., 2011; Gomez‐Uchida et al., 2013). Further development of these concepts, considering also other theoretical models and unequal contribution from subpopulations, suggests that subdivision in natural populations may lead rather to a decrease than to an increase of the global *N*_*e*_, also depending on the geographical scale of the study area (see Neel et al., 2013; Wang & Caballero, 1999).

Although population heterogeneity has been considered in latest empirical studies on estimating *N*_*e*_ in natural populations (Fraser et al., 2007; Gomez‐Uchida, Palstra, Knight, & Ruzzante, 2013; Hindar, Tufto, Sættem, & Balstad, 2004; Kuparinen et al., 2010; Laikre, Olsson, Jansson, Hössjer, & Ryman, 2016; Nunney, 1999; Palstra & Ruzzante, 2011; Ruzzante et al., 2016; Tufto & Hindar, 2003), there is still a lack of empirical data exploring the interplay of the global *N*_*e*_ (or meta‐*N*_*e*_) of structured populations and the sum of local or subpopulation *N*_*e*_ (∑*N*_*e*(s)_). Especially in studies operating on a large scale, these parameters may be underestimated due to mixture LD caused by combining more than one gene pool (England et al., 2010; Palstra & Ruzzante, 2011; Waples & England, 2011; Wang & Caballero, 1999; Whitlock and Barton, 1997). Over the last decades, numerous wildlife populations in Europe have been recovering (Chapron, et al. 2014), leading to increased admixture among formerly separated populations (Hagen, Kopatz, Aspi, Kojola, & Eiken, 2015). Large, terrestrial mammals often show genetic structure due to previous fragmentation and isolation (e.g., Norman, Street, & Spong, 2013; Schregel et al., 2015; Stronen et al., 2013). In such cases, not considering population admixture may underestimate *N*_*b*_ due to increased drift LD caused by the growing number of parents responsible for local samples (Waples & England, 2011).

We have used the recovering Finnish brown bear population (*Ursus arctos*) as a natural model system to estimate the effective number of breeders (${\hat{N}}_{b}$) and effective population size (${\hat{N}}_{e}$) under rapidly decreasing population structure and increasing admixture. The Finnish brown bear population underwent significant changes within only 1.5 generations due to demographic growth, immigration from Russia, and range expansion (Hagen et al., 2015; Kopatz et al., 2014). Specifically, the degree of population differentiation between the northern and southern genetic cluster decreased rapidly from *F*_ST_ = 0.051 in 1996 to *F*_ST_ = 0.014 in 2010, while the estimated number of migrants per generation between them increased from 1.6 to 3.6. Also, the pattern of isolation by distance debilitated within this time. All changes detected suggest merging of the two genetic clusters during population recovery (see Hagen et al., 2015), thus creating an opportunity to estimate the temporal trends of ${\hat{N}}_{b}$ and ${\hat{N}}_{e}$ during rapidly decreasing population structure in a natural population. We tracked ${\hat{N}}_{e}$ through the latest phase of population recovery of the Finnish brown bear, for individuals born between 1993 and 2010, both with and without accounting for substructure and admixture to investigate their effect on the estimates between the two approaches. We hypothesize that the difference between these approaches, as suggested by theoretical studies and simulations (Antao et al., 2011; England et al., 2010; Waples & England, 2011), disappears as the population recovers and substructure diminishes (Hagen et al., 2015).

## 2 MATERIAL AND METHODS

**Figure 1:**
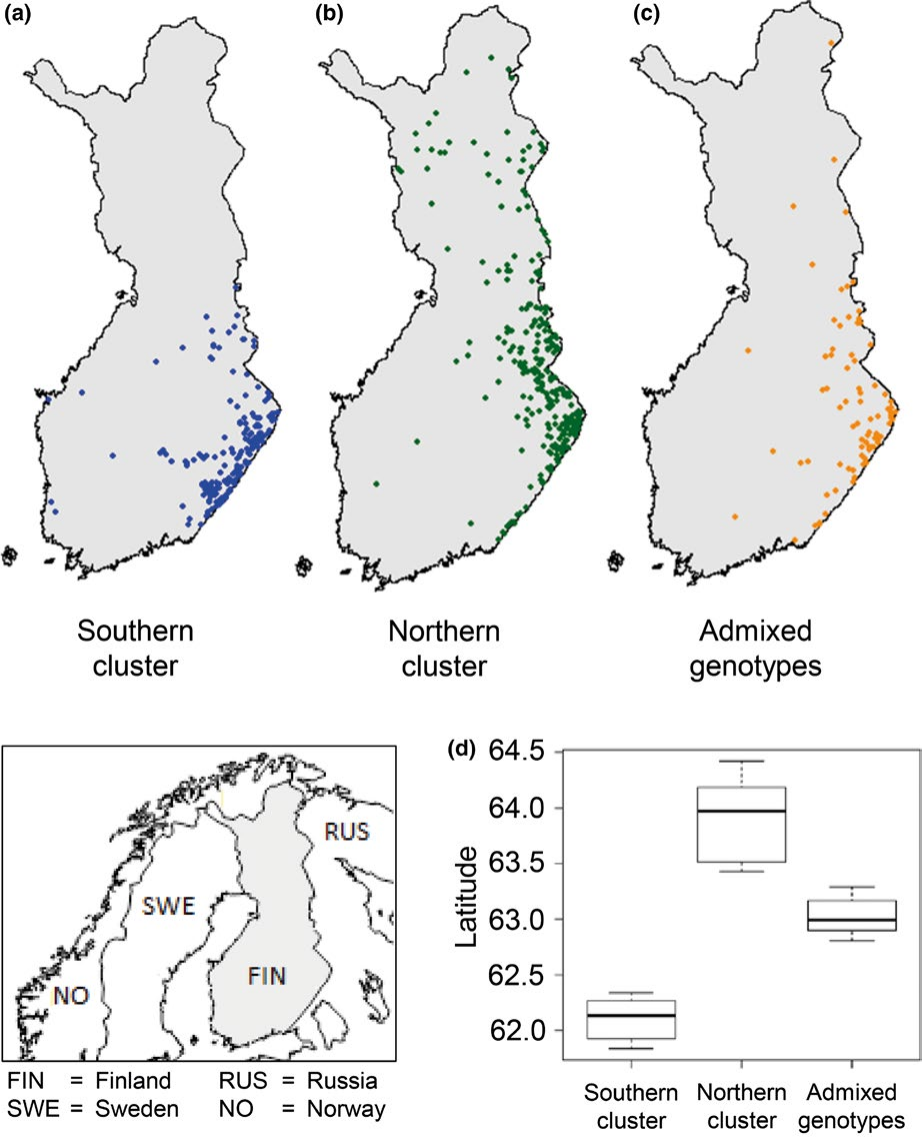
Individual genotypes of brown bears born between 1993 and 2010 and legally harvested in Finland, their sampling location and genotypes assigned with the program STRUCTURE (Pritchard et al., 2000) and a membership coefficient *q *>* *0.7 to the (a) southern genetic cluster (*N *=* *230) and (b) northern cluster (*N *=* *316) as well as (c) not clearly assigned, admixed genotypes, with a membership coefficient below the threshold of *q *<* *0.7 (*N *=* *74) for each of the two clusters. (d) Mean of the average geographical latitudes of brown bears assigned to the southern and northern genetic cluster as well as the mean of the average latitudes of the individuals with admixed genotypes sampled in Finland for each 3‐years birth group, as it was used as predictor variable for all further statistical analysis

We used georeferenced data of 710 brown bears (252 females and 458 males) legally harvested in Finland from 1996 to 2010. The age of each brown bear was estimated using tooth samples (Craighead, Craighead, & McCutchen, 1970) by Matson's Laboratory (LLC, Milltown, Montana). Individuals were genotyped with 12 validated microsatellite markers and assigned to either the southern or northern genetic cluster earlier (see Hagen et al., 2015; Kopatz et al., 2014). We pooled the data into six birth groups containing 3 years of genotype data of brown bears born in these years to increase sample sizes covering a period from 1993 to 2010 (Figure 1; average sample size per birth group *N *= 88, *SD* = 39.4). The oldest brown bears were born in 1977, but we used individuals born between 1993 and 2010 for this analysis due to too low and varying sample sizes prior to that period (89 individuals born between 1977 and 1992). We used the linkage disequilibrium estimator LDNE (Waples & Do, 2008) to estimate the raw *N*_*b*_ (raw ${\hat{N}}_{b}$) and calculated the criterion for the exclusion of rare alleles as suggested by Waples and Do (2010) using the formula 1/(2 x *N*) < *P*_crit_ < 1/*N*. Raw N¨b was estimated both with and without accounting for the gradually increasing admixture and decreasing differentiation between the southern and northern cluster as recovery proceeded using a membership value (*q*) ≥ 0.7 (Hagen et al., 2015; Kopatz et al., 2014) as threshold for individual cluster assignment by the program structure (Pritchard, Stephens, & Donnelly, 2000). Raw estimates (raw ${\hat{N}}_{b}$) were subsequently adjusted (${\hat{N}}_{b_{\left(\mathrm{adj}\right)}}$) using the method and formula by Waples et al. (2014) including two life‐history traits available, from the North American brown bear, the grizzly: age at first reproduction (α) and adult life span (AL):

(1) $${\hat{N}}_{b_{\left(\mathrm{adj}\right)}}=\frac{\text{raw}\;{\hat{N}}_{b}}{1.103\;-\;0.245\;\times \;\text{log}(\frac{\text{AL}}{\alpha })}$$

After, we used the adjusted estimate of effective number of breeders (${\hat{N}}_{b_{\left(\mathrm{adj}\right)}}$) to estimate the adjusted effective population size (${\hat{N}}_{e_{\left(\mathrm{adj}\right)}}$) using the same, two traits by applying the following formula (Waples et al., 2014):

(2) $${\hat{N}}_{e_{\left(\mathrm{adj}\right)}}=\frac{{\hat{N}}_{b_{\left(\mathrm{adj}\right)}}}{0.485\;+\;0.758\;\times \;\text{log}(\frac{\text{AL}}{\alpha })}$$

We tested for correlation of each category of ${\hat{N}}_{b_{\left(\mathrm{adj}\right)}}$ with the increasing minimum census number (*N*_*c*_) of brown bears in the country, which is annually estimated based on brown bear observations and large carnivore contact persons in the different hunting districts (Wikman, 2010). We also tested whether the estimates have a relationship with latitudinal expansion of each genetic cluster.

Due to too low sample sizes of admixed genotypes in each birth group, the temporal raw ${\hat{N}}_{b}$ and ${\hat{N}}_{b_{\left(\mathrm{adj}\right)}}$ for this group was inferred indirectly by comparing estimates from separate analyses that either included or excluded them. Therefore, we estimated raw ${\hat{N}}_{b}$, ${\hat{N}}_{b_{\left(\mathrm{adj}\right)}}$, and ${\hat{N}}_{e_{\left(\mathrm{adj}\right)}}$ for admixed genotypes also by pooling them across the last 10 years of our study period, which corresponds to the generation length of brown bears (Tallmon, Bellemain, Swenson, & Taberlet, 2004; Waples et al., 2014). For comparison, this was also carried out for all genotypes and for each genetic cluster separately. In this way, we obtained a direct estimate of the relative influence of admixed genotypes on ${\hat{N}}_{b_{\left(\mathrm{adj}\right)}}$ and ${\hat{N}}_{e_{\left(\mathrm{adj}\right)}}$.

We also scrutinized the available data if possible demographic changes could explain the changes in the Finnish brown bear population by calculating the proportion of males and females and the proportion of brown bears in reproductive age above 4 years of age (Støen, Zedrosser, Wegge, & Swenson, 2006) across the study period. Statistical tests were performed with R (R Core Development Team, 2017).

## 3 RESULTS

**Figure 2:**
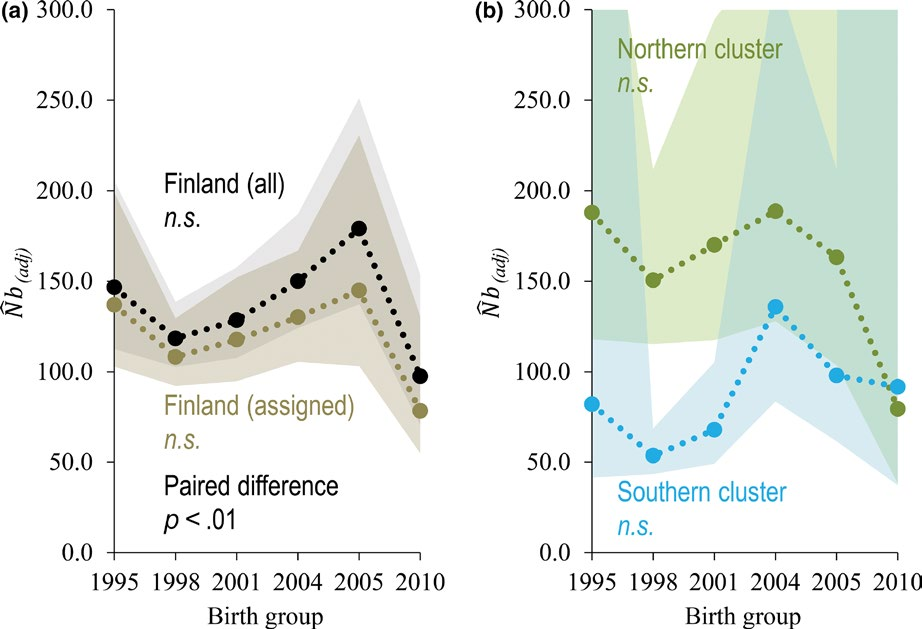
Six 3‐year birth groups of brown bears born between 1993 and 2010 in Finland: (a) adjusted estimates of the number of breeders (${\hat{N}}_{b_{\left(\mathrm{adj}\right)}}$) for all analyzed genotypes from Finnish brown bear population (black) versus only clearly assigned genotypes with a membership value *q *≥* *0.7 without admixed genotypes (brown). (b) ${\hat{N}}_{b_{\left(\mathrm{adj}\right)}}$ for the genetic clusters (green = northern cluster, blue = southern cluster) including only genotypes assigned with a membership value *q *≥* *0.7 for each genetic cluster. The shaded areas represent 95% confidence intervals. See Tables 1 and 2 for further results

The temporal trend of ${\hat{N}}_{b_{\left(\mathrm{adj}\right)}}$ for the Finnish brown bear population when all genotypes were pooled (i.e., meta‐${\hat{N}}_{b}$), including also admixed genotypes, displayed an increasing trend across time (harmonic mean (HM) ${\hat{N}}_{b_{\left(\mathrm{adj}\right)}}$ ≈ 131.7, Tables 1 and 2, Figure 2a; see Table S1 for the raw ${\hat{N}}_{b}$), however, with a drop of the estimates for the last birth group (Figure 2a). Excluding admixed genotypes and using only unambiguously assigned genotypes for the analyses resulted in significantly lower values for the estimates (paired *t* test, *t* = 4.51, *df* = 5, *p *< .01), but a similar trajectory of ${\hat{N}}_{b_{\left(\mathrm{adj}\right)}}$ across time (HM ${\hat{N}}_{b_{\left(\mathrm{adj}\right)}}$ ≈ 114.5, Tables 1 and 2, Figure 2a). The calculation from ${\hat{N}}_{b_{\left(\mathrm{adj}\right)}}$ to ${\hat{N}}_{e_{\left(\mathrm{adj}\right)}}$ approximately doubled the estimates (HM ${\hat{N}}_{e_{\left(\mathrm{adj}\right)}}$ ≈ 272.1; Table 1).

**Table 1:** Brown bears born between 1993 and 2010 in Finland separated into six 3‐year birth groups including minimum census sizes (*Nc*_MINIMUM_) based on observations (see Material and Methods), samples sizes (*N*) and adjusted estimates of effective number of breeders (${\hat{N}}_{b_{\left(\mathrm{adj}\right)}}$) using two life‐history traits (life span and age at first reproduction; Waples et al., 2014; see Material and Methods), based on the raw ${\hat{N}}_{b}$ (see Table S1) from the linkage disequilibrium‐based estimation with the program LDNE (Waples & Do, 2008) and adjusted estimates of effective populations size (${\hat{N}}_{e_{\left(\mathrm{adj}\right)}}$) for the whole Finnish brown bear population (_FINLAND_), unambiguously assigned genotypes only (_FINLAND (ASSIGNED)_) as well as for the southern (_SOUTH_) and northern (_NORTH_) genetic cluster

Birth group	Minimum population size (*N*_*c*_)	Sample sizes (*N*)				Adjusted estimates of effective number of breeders ${\hat{N}}_{b_{\left(\mathrm{adj}\right)}}$												Adjusted estimates of effective population sizes ${\hat{N}}_{e_{\left(\mathrm{adj}\right)}}$			
	*Nc*_MINIMUM_	*N*_FINLAND_	*N*_SOUTH_	*N*_NORTH_	*N*_ADMIXED_	${\hat{N}}_{b_{\left(\mathrm{adj}\right)}}$_FINLAND_	95% CI		${\hat{N}}_{b_{\left(\mathrm{adj}\right)}}$_FINLAND (ASSIGNED)_	95% CI		${\hat{N}}_{b_{\left(\mathrm{adj}\right)}}$_SOUTH_	95% CI		${\hat{N}}_{b_{\left(\mathrm{adj}\right)}}$_NORTH_	95% CI		${\hat{N}}_{e_{\left(\mathrm{adj}\right)}}$_FINLAND_	${\hat{N}}_{e_{\left(\mathrm{adj}\right)}}$_FINLAND (ASSIGNED)_	${\hat{N}}_{e_{\left(\mathrm{adj}\right)}}$_SOUTH_	${\hat{N}}_{e_{\left(\mathrm{adj}\right)}}$_NORTH_
1993–1995	686	79	49	25	5	146.6	112.1	205.4	136.9	102.4	198.9	82.0	41.1	622.2	187.8	117.6	423.6	302.8	282.8	169.8	387.9
1996–1998	783	158	80	60	18	118.4	102.3	138.6	108.1	91.8	129.5	53.6	43.1	68.6	150.5	114.9	211.9	244.7	223.6	111.1	310.9
1999–2001	845	116	61	43	12	128.4	107.2	157.6	117.7	94.5	152.1	67.9	48.8	105.3	170.1	117.1	294.5	265.5	243.2	140.6	351.3
2002–2004	815	127	63	45	19	149.9	123.7	187.0	130.1	105.1	166.9	135.8	83.1	321.7	188.6	127.5	340.7	309.8	268.8	280.6	389.6
2005–2007	840	96	46	40	10	178.9	137.0	251.0	144.9	102.8	230.5	97.9	61.3	211.9	163.2	103.2	353.0	369.6	299.3	202.5	337.1
2008–2010	1,070	45	17	18	10	97.4	69.8	153.7	78.3	54.4	130.5	91.6	36.9	∞	79.4	37.5	7,920.2	201.5	162.1	189.6	164.3
Harmonic mean	825.3	88.1	41.2	32.8	10.1	131.7			114.5			81.0			143.3			272.1	236.7	167.7	296.1
*SD*	126.9	39.4	21.2	15.0	5.3																

**Table 2:** Correlations of the temporal trends for the estimates of the number of effective breeders (${\hat{N}}_{b_{\left(\mathrm{adj}\right)}}$) of six birth groups across the study period from 1993 to 2010 of the Finnish brown bear population. We used the whole dataset (${\hat{N}}_{b_{\left(\mathrm{adj}\right)}}$_FINLAND_), only clearly assigned genotypes (${\hat{N}}_{b_{\left(\mathrm{adj}\right)}}$_FINLAND (ASSIGNED)_) and its northern (${\hat{N}}_{b_{\left(\mathrm{adj}\right)}}$_NORTH_) and southern (${\hat{N}}_{b_{\left(\mathrm{adj}\right)}}$_SOUTH_) genetic clusters and the absolute difference of the latter (${\hat{N}}_{b_{\left(\mathrm{adj}\right)}}$_NORTH_)‐( ${\hat{N}}_{e_{\left(\mathrm{adj}\right)}}$_SOUTH_). We correlated the results for each birth group against time (using the last year of the respective birth group). Further, the table includes correlations of the trends on the relative proportion of the number of breeders (Proportion of ${\hat{N}}_{b_{\left(\mathrm{adj}\right)}}$_NORTH_) of the northern genetic cluster and the trend of the proportion of admixed genotypes (Proportion _ADMIXED_) over the study period (using the last year of the birth group)

Model/Response	Intercept (95% CI)	Predictor/Cohort (95% CI)	*R* ^2^	*t*‐value	Significance
${\hat{N}}_{b_{\left(\mathrm{adj}\right)}}$_FINLAND_ vs. time	140.88 (59.38, 222.37)	−0.41 (−7.38, 6.57)	−0.24	−0.16	n.s.
${\hat{N}}_{b_{\left(\mathrm{adj}\right)}}$_FINLAND (ASSIGNED)_ vs. time	136.35 (72.10, 200.60)	−1.62 (−7.12, 3.88)	−0.01	−0.82	n.s.
${\hat{N}}_{b_{\left(\mathrm{adj}\right)}}$_NORTH_ vs. time	205.15 (115.05, 295.22)	−4.62 (−12.33, 3.09)	0.26	−1.67	n.s.
${\hat{N}}_{b_{\left(\mathrm{adj}\right)}}$_SOUTH_ vs. time	63.24 (−9.13, 135.61)	2.37 (−3.82, 8.57)	0.03	1.06	n.s.
${\hat{N}}_{b_{\left(\mathrm{adj}\right)}}$_NORTH_ *vs*. ${\hat{N}}_{b_{\left(\mathrm{adj}\right)}}$_SOUTH_	141.91 (78.58, 205.24)	−7.67 (−12.47, −2.87)	0.79	−4.44	*p* < .05
Proportion of ${\hat{N}}_{b_{\left(\mathrm{adj}\right)}}$_NORTH_ vs. time	0.80 (0.64, 0.96)	−0.02 (−0.03, −0.002	0.65	−3.21	*p* < .05
Proportion _ADMIXED_ vs. time	0.05 (−0.05, 0.14)	0.008 (−4.53, 0.02)	0.54	2.63	n.s., *p *= .059

**Figure 3:**
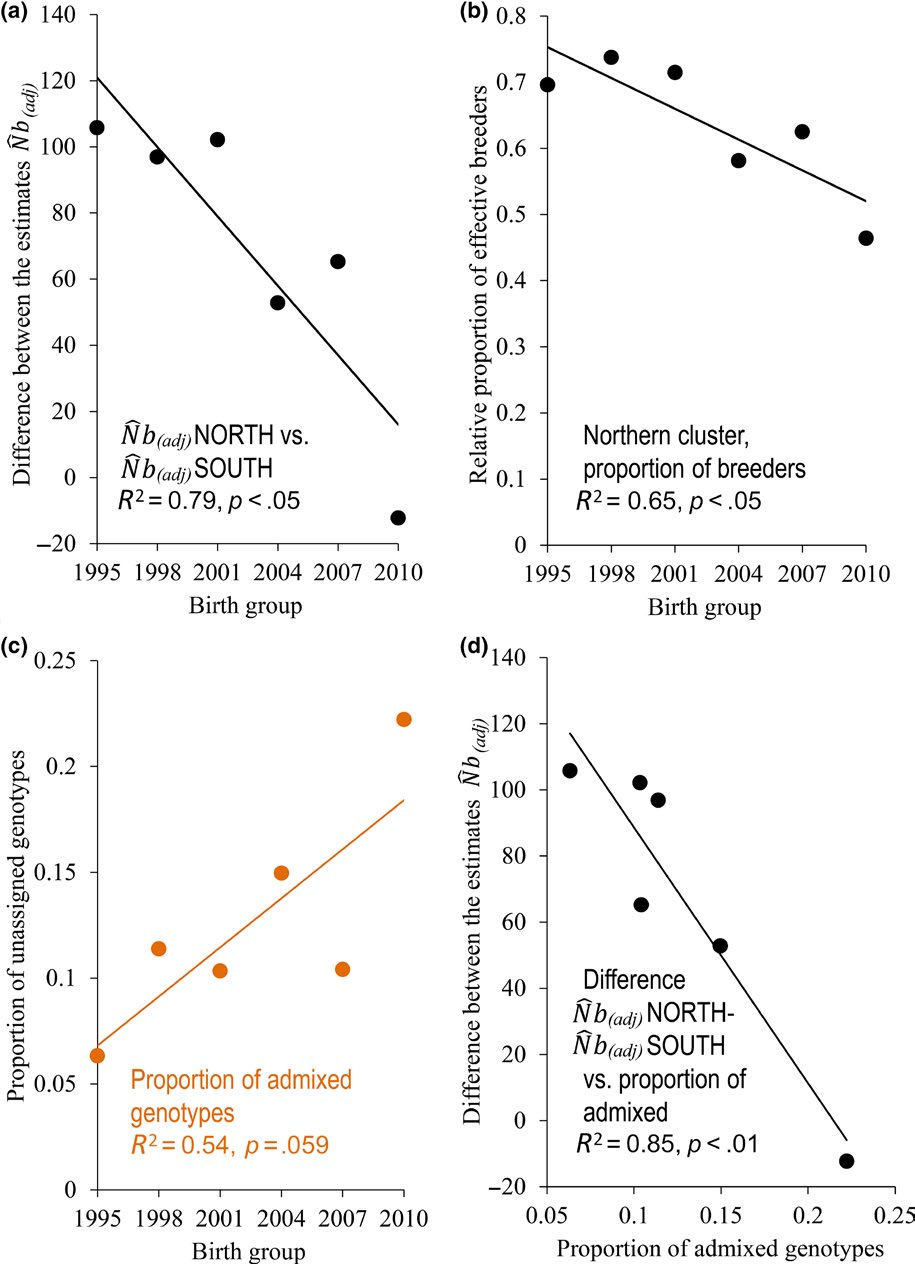
Six 3‐year birth groups of brown bears born between 1993 and 2010 in Finland: (a) The difference of the estimates of the number of breeders in absolute values of the northern and southern genetic cluster across the study period. The number of effective breeders was based on the adjusted estimates (${\hat{N}}_{b_{\left(\mathrm{adj}\right)}}$) (see Fig. 2b and Results). (b) The proportion of the number of effective breeders (here northern cluster). (c) Proportion of admixed and unassigned genotypes over the period with a membership value *q *<* *0.7 (orange). (d) The difference between ${\hat{N}}_{b_{\left(\mathrm{adj}\right)}}$ of the northern and southern genetic cluster correlated against the proportion of admixed genotypes. Results here are presented by the last year of the birth group. See Tables 1 and 2 for more statistical results

The absolute values and temporal trajectory of ${\hat{N}}_{b_{\left(\mathrm{adj}\right)}}$ depended strongly on the degree of genetic substructure. Notably, ${\hat{N}}_{b_{\left(\mathrm{adj}\right)}}$ for the northern cluster alone was higher (HM ${\hat{N}}_{b_{\left(\mathrm{adj}\right)}}$ ≈ 143.3) than for the whole Finnish population (HM ${\hat{N}}_{b_{\left(\mathrm{adj}\right)}}$ = 131.7; Tables 1 and 2; Figure 2b), but decreased toward the end of the study period after a peak of ${\hat{N}}_{b_{\left(\mathrm{adj}\right)}}$ = 188.6 in birth group 2002–2004 (Tables 1 and 2; Figure 2b). In comparison, ${\hat{N}}_{b_{\left(\mathrm{adj}\right)}}$ for the southern cluster was relatively low (HM ${\hat{N}}_{b_{\left(\mathrm{adj}\right)}}$ ≈ 81) for the early birth groups from 1995 to 2001 and relatively high for the three final birth groups (Tables 1 and 2; Figure 2b). Thus, the estimates for the two clusters converged over time as they merged (*P *<* *0.05; Table 2; Figure 3a). The sum of the two estimates (${\hat{N}}_{b_{\left(\mathrm{adj}\right)}}$ = 224.3, i.e., (∑${\hat{N}}_{e_{\left(s\right)}}$) was 70.3% larger than the estimate for the whole Finnish brown bear population (HM ${\hat{N}}_{b_{\left(\mathrm{adj}\right)}}$ ≈ 131.7; Table 1). Similar results were found for ${\hat{N}}_{e_{\left(\mathrm{adj}\right)}}$, which was consistently larger than ${\hat{N}}_{b_{\left(\mathrm{adj}\right)}}$ (Table 1). As substructure gradually declined, the proportion of effective breeders in the southern cluster went from 30% to 54%, while the northern cluster went from 70% to 46% (*p *< .05; Table 2; Figure 3b). In the same time, the proportion of admixed individuals in the area between the two genetic clusters increased from 0.06 to 0.22 (*p *= .059; Table 2; Figure 3c). Thus, the proportion of breeders between the clusters equalized as admixture increased (*p *< .05; Table 3). Also, the difference of the estimates of ${\hat{N}}_{b_{\left(\mathrm{adj}\right)}}$ between the northern and southern cluster decreased with the proportion of admixed genotypes (*p *< .01; Table 3; Figure 3d).

**Table 3:** Correlations of the trends for the adjusted estimates of the number of effective breeders (${\hat{N}}_{b_{\left(\mathrm{adj}\right)}}$) of the Finnish brown bear population across the study period from 1993 to 2010 as a whole (${\hat{N}}_{b_{\left(\mathrm{adj}\right)}}$_FINLAND_), clearly assigned genotypes only (${\hat{N}}_{b_{\left(\mathrm{adj}\right)}}$_FINLAND (ASSIGNED)_) and the identified northern (${\hat{N}}_{b_{\left(\mathrm{adj}\right)}}$_NORTH_) and southern (${\hat{N}}_{b_{\left(\mathrm{adj}\right)}}$_SOUTH_) genetic clusters correlated against the change of the average latitude of the northern and southern genetic clusters. Further, correlations of the adjusted estimates of the number of effective breeders (${\hat{N}}_{b_{\left(\mathrm{adj}\right)}}$) against the estimations of the minimum census size (*N*_*c*__MINIMUM_) of brown bears in Finland, as well as correlation of the proportion of breeders in the northern cluster (Proportion ${\hat{N}}_{b_{\left(\mathrm{adj}\right)}}$_NORTH_ versus admixed genotypes (Proportion _ADMIXED_), admixed genotypes (Proportion _ADMIXED_) versus the minimum census size (*N*_*c*__MINIMUM_) and the difference of ${\hat{N}}_{b_{\left(\mathrm{adj}\right)}}$ versus the admixed genotypes (Proportion _ADMIXED_)

Model/Response	Predictor/Cohort (95% CI)	*R* ^2^	*t*‐value	Significance
${\hat{N}}_{b_{\left(\mathrm{adj}\right)}}$_FINLAND_ vs. Latitude_NORTH_	13.82 (−80.82, 108.46)	−0.20	0.41	n.s.
${\hat{N}}_{b_{\left(\mathrm{adj}\right)}}$_FINLAND (ASSIGNED)_ vs. Latitude_NORTH_	−2.32 (−84.26, 79.61)	−0.25	−0.08	n.s.
${\hat{N}}_{b_{\left(\mathrm{adj}\right)}}$_NORTH_ vs. Latitude_NORTH_	−34.50 (164.37, 95.37)	−0.10	−0.74	n.s.
${\hat{N}}_{b_{\left(\mathrm{adj}\right)}}$_FINLAND_ vs. Latitude_SOUTH_	37.55 (144.50, 219.60)	−0.16	0.57	n.s.
${\hat{N}}_{b_{\left(\mathrm{adj}\right)}}$_FINLAND (ASSIGNED)_ vs. Latitude_SOUTH_	2.93 (−157.81, 163.68)	−0.25	0.05	n.s.
${\hat{N}}_{b_{\left(\mathrm{adj}\right)}}$_SOUTH_ vs. Latitude_SOUTH_	118.14 (22.56, 213.71)	0.68	3.43	*p* < .05
${\hat{N}}_{b_{\left(\mathrm{adj}\right)}}$_FINLAND_ vs. *Nc*_MINIMUM_	−0.12 (−0.38, 0.14)	0.12	−1.32	n.s.
${\hat{N}}_{b_{\left(\mathrm{adj}\right)}}$_FINLAND (ASSIGNED)_ vs. *Nc*_MINIMUM_	−0.15 (−0.31, 0.02)	0.50	−2.43	n.s., *p* = .071
${\hat{N}}_{b_{\left(\mathrm{adj}\right)}}$_NORTH_ vs. *Nc*_MINIMUM_	−0.29 (−0.49, −0.08)	0.74	−3.94	*p* < .05
${\hat{N}}_{b_{\left(\mathrm{adj}\right)}}$_SOUTH_ vs. *Nc*_MINIMUM_	0.03 (−0.28, 0.34)	−0.23	0.27	n.s.
Proportion of ${\hat{N}}_{b_{\left(\mathrm{adj}\right)}}$_NORTH_ vs. proportion_ADMIXED_	−1.63 (−2.92, −0.35)	0.70	−3.52	*p* < .05
Proportion_ADMIXED_ vs. *Nc*_MINIMUM_	0.0004 (0.0002, 0.0006)	0.80	4.63	*p* < .01
Difference of ${\hat{N}}_{b_{\left(\mathrm{adj}\right)}}$_NORTH and SOUTH_ vs. proportion_ADMIXED_	−773.53 (−1168.43, −378.63)	0.85	−5.44	*p* < .01

Genotypes of the southern and northern clusters showed substantial geographical overlap (see Figure 1). Despite this overlap, the average latitude of both genetic groups differed, and the admixed genotypes were mainly sampled in the area where both clusters meet (Figure 1d). Based on the birth year of individuals, the average latitude of the genetic clusters shifted northwards over time as the population expanded (southern cluster, *p *< .05; Table S2; Figure S1a; northern cluster, *p *= .068; Table S2; Fig. S1a). Temporal increase of ${\hat{N}}_{b_{\left(\mathrm{adj}\right)}}$ for the southern cluster was correlated with its northwards expansion (*P *<* *0.05; Table 3; Fig. S1b), while other relationships of ${\hat{N}}_{b_{\left(\mathrm{adj}\right)}}$ with the observed range expansion were not significant (Table 3).

${\hat{N}}_{b_{\left(\mathrm{adj}\right)}}$ for whole Finland did not show any significant correlation with the trends of the estimated minimum population size *N*_*c*_ (Table 3). The same applied to the trends of ${\hat{N}}_{b_{\left(\mathrm{adj}\right)}}$ for the southern genetic cluster, while ${\hat{N}}_{b_{\left(\mathrm{adj}\right)}}$ for the northern cluster showed a significant correlation with the estimated minimum *N*_*c*_ (*P *<* *0.05; Table 3; Fig. S1c). Notably, the proportion of unassigned and admixed genotypes correlated with the minimum census size *N*_*c*_ (*P *<* *0.01; Table 3).

**Figure 4:**
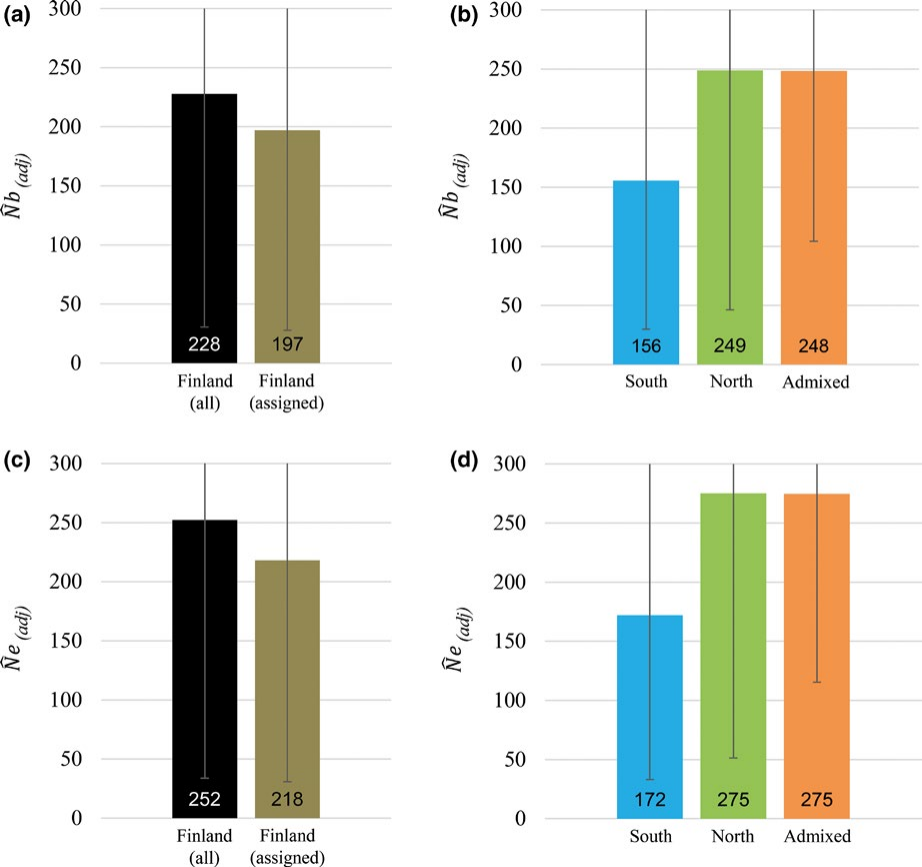
Estimates of ${\hat{N}}_{b_{\left(\mathrm{adj}\right)}}$ and ${\hat{N}}_{e_{\left(\mathrm{adj}\right)}}$ of one generation in the Finnish brown bear population including genotypes of individuals born between 2000 and 2010: (a) ${\hat{N}}_{b_{\left(\mathrm{adj}\right)}}$ of all genotypes from Finland (black) and ${\hat{N}}_{e_{\left(\mathrm{adj}\right)}}$ of only unambiguously assigned genotypes from Finland (brown; membership value *q *≥* *0.7); (b) ${\hat{N}}_{e_{\left(\mathrm{adj}\right)}}$ for the southern (blue), northern (green), and unassigned (admixed) genotypes (orange); (c) ${\hat{N}}_{e_{\left(\mathrm{adj}\right)}}$ of all genotypes from Finland (black) and ${\hat{N}}_{e_{\left(\mathrm{adj}\right)}}$ of only unambiguously assigned genotypes from Finland; (d) ${\hat{N}}_{e_{\left(\mathrm{adj}\right)}}$ for the southern (blue), northern (green), and unassigned (admixed) genotypes (orange)

Genotypes were pooled across the years 2000–2010 (representing one generation, Figure 4) to estimate ${\hat{N}}_{b_{\left(\mathrm{adj}\right)}}$ and ${\hat{N}}_{e_{\left(\mathrm{adj}\right)}}$ for the group of admixed genotypes directly. These results showed a substantially higher influence of these genotypes than the indirect approach suggested (Figure 4). Separate estimates for the southern cluster (${\hat{N}}_{b_{\left(\mathrm{adj}\right)}}$ = 156; ${\hat{N}}_{e_{\left(\mathrm{adj}\right)}}$ = 172), northern cluster (${\hat{N}}_{b_{\left(\mathrm{adj}\right)}}$ = 249; ${\hat{N}}_{e_{\left(\mathrm{adj}\right)}}$ = 275), and group of admixed genotypes (${\hat{N}}_{b_{\left(\mathrm{adj}\right)}}$ = 248; ${\hat{N}}_{e_{\left(\mathrm{adj}\right)}}$ = 275) summed up to ∑${\hat{N}}_{b_{\left(\mathrm{adj}\right)}}$ = 653 and ∑${\hat{N}}_{e_{\left(\mathrm{adj}\right)}}$ = 722 which exceeded the estimates which did not account for population substructure (${\hat{N}}_{b_{\left(\mathrm{adj}\right)}}$ = 228; ${\hat{N}}_{e_{\left(\mathrm{adj}\right)}}$ = 252). Consistently, ${\hat{N}}_{b_{\left(\mathrm{adj}\right)}}$ was higher than the harmonic means across birth groups in the temporal analyses. Results of ${\hat{N}}_{e_{\left(\mathrm{adj}\right)}}$ on the other hand were lower than the harmonic means across birth groups. Again, using the last ten years of our study period, the results of ${\hat{N}}_{b_{\left(\mathrm{adj}\right)}}$ were consistently lower than for ${\hat{N}}_{e_{\left(\mathrm{adj}\right)}}$.

**Figure 5:**
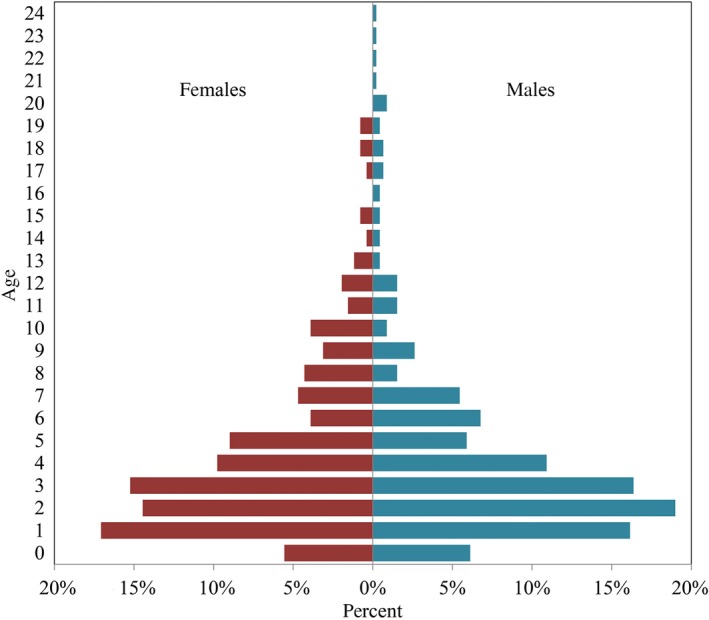
Demographic pyramid of the brown bear harvest data from Finland from 1996 to 2010

The age distribution across individuals displayed the pattern of a growing population, consisting mainly of younger and fewer old individuals (Figure 5, Fig. S2a), suggesting a sample representative of the ongoing population recovery. Overall, the harvest data became more male‐biased over time (*p *< .05; Fig. S2b). However, there was no difference between clusters that could potentially cause a bias in our estimations. No trends were detected for brown bears younger than three and older than 4 years of age (Fig. S2c–d). No correlations with sample size were detected.

## 4 DISCUSSION

We applied the single‐sample approach LDNE (Waples & Do, 2008) to assess the impact of genetic substructure and admixture on ${\hat{N}}_{b_{\left(\mathrm{adj}\right)}}$ (Waples et al., 2014) in the naturally recovering brown bear population of Finland, using individuals born between 1993 and 2010. We found ${\hat{N}}_{b_{\left(\mathrm{adj}\right)}}$ for the entire Finnish brown bear population to be lower than the sum of the separate estimates of the two genetic clusters. Also, we found that admixture constantly increased ${\hat{N}}_{b_{\left(\mathrm{adj}\right)}}$. When estimating ${\hat{N}}_{b_{\left(\mathrm{adj}\right)}}$ separately, the northern cluster showed the highest estimates, although with a decrease during the last part of the study period. Contrary, for the southern cluster, ${\hat{N}}_{b_{\left(\mathrm{adj}\right)}}$ was initially lower and increased across time. At the end of the study period, the two clusters had nearly merged and showed almost equal ${\hat{N}}_{b_{\left(\mathrm{adj}\right)}}$. ${\hat{N}}_{e_{\left(\mathrm{adj}\right)}}$ results were larger than ${\hat{N}}_{b_{\left(\mathrm{adj}\right)}}$ and likely reflective of brown bear populations inhabiting both Finland and northwestern Russia.

Effects of both, increased mixture LD and reduced drift LD, (Waples & England, 2011) may be displayed in our results. When pooling both subpopulations for analysis, ${\hat{N}}_{b_{\left(\mathrm{adj}\right)}}$ displayed a downward effect compared to the separate estimates of the two genetic clusters, which according to theory may be due to increased mixture LD (Whitlock and Barton, 1996; Wang & Caballero, 1999; England et al., 2010; Waples & England, 2011). Also, ${\hat{N}}_{b_{\left(\mathrm{adj}\right)}}$ for whole Finland was lower than for the northern cluster. At the same time, a reduction of drift LD may have caused an upward effect on the estimates due to increasing admixture between the two subpopulations (England et al., 2010; Waples & England, 2011). All analyses including admixed genotypes showed higher ${\hat{N}}_{b_{\left(\mathrm{adj}\right)}}$ than those excluding them. Our estimates are reflective of the dissolving genetic substructure, increased gene flow between clusters, and decreasing LD among loci over time as described previously in Hagen et al. (2015).

We estimated ${\hat{N}}_{b}$ and ${\hat{N}}_{e}$ in an open and natural system, where immigrants can have a positive effect on *N*_*b*_ by increasing genetic variation (Charlesworth, 2009). In our case, the high immigration from Russia (Kopatz et al., 2014) probably increased the estimates due to decreased LD and reduced drift LD as more parents would produce the local sample (Waples & England, 2011). Increased immigration may have also caused the temporal increase of male bears in the data, as dispersal in brown bears is male‐biased (Støen, Zedrosser, Sæbø, & Swenson, 2006; Zedrosser, Støen, Sæbo, & Swenson, 2007). Our results indicate that the recovery of the Finnish brown bear population is likely driven by immigration of individuals from Russia into the southern cluster, a route that other studies have suggested earlier (Hagen et al., 2015; Keis et al., 2012; Kopatz et al., 2014). This is supported by the relationship of the trend of the ${\hat{N}}_{b_{\left(\mathrm{adj}\right)}}$ for the southern cluster with its northward range expansion. Currently, there are no reliable estimates on the population size of brown bears in the regions in Russia neighboring Finland which would allow for better comparisons.

The overall trend of ${\hat{N}}_{b_{\left(\mathrm{adj}\right)}}$ for Finland did not follow the demographic recovery due to a drop of ${\hat{N}}_{b_{\left(\mathrm{adj}\right)}}$ in the latest birth group. Although LDNE includes a correction for small sample sizes (*N *<* *30; Waples 2006), the latter estimates may be biased by consisting of only local, young individuals (Baalsrud et al., 2014). These may not be representative for the population and ongoing demographic recovery compared to the brown bears in the older birth groups. Further, it is also possible that increased immigration of brown bears from Russia during the latest phase (Hagen et al., 2015; Kopatz et al., 2014) may have influenced estimate precision by leading to large confidence intervals (Baalsrud et al., 2014). Throughout the study period, the southern cluster showed a substantial increase of the relative proportion of effective breeders, while the proportion for the northern cluster decreased, resulting in approximately equal proportions of effective breeders from the two clusters.

Waples and England, (2011) showed that when the migration rate increases, estimations based on local data rather represent global or metapopulation *N*_*e*_. Thus, our *N*_*e*_‐estimates are most likely influenced by the fact that the Finnish brown bear population originates and is part of the Russian population (Kopatz et al., 2012 & 2014) and therefore may be considered as an upper global estimate for the region of Finland and northwestern Russia. In that light, although results of ${\hat{N}}_{b_{\left(\mathrm{adj}\right)}}$ were larger than for ${\hat{N}}_{e_{\left(\mathrm{adj}\right)}}$, the estimates appear to be rather low, considering the assumption that Russia houses the largest brown bear population in the world.

The results of ${\hat{N}}_{e_{\left(\mathrm{adj}\right)}}$ when pooling genotypes of the last decade of our study period (representing one generation) differed from the harmonic mean of the birth groups. Here, ${\hat{N}}_{b_{\left(\mathrm{adj}\right)}}$ and ${\hat{N}}_{e_{\left(\mathrm{adj}\right)}}$ of the admixed genotypes were notably larger, indicating a substantial contribution to the pool of breeders. The sum of the three estimates, ${\hat{N}}_{b_{\left(\mathrm{adj}\right)}}$ and ${\hat{N}}_{e_{\left(\mathrm{adj}\right)}}$ for southern and northern cluster as well as for admixed individuals, were nearly three times as large as the respective estimates for the undivided dataset (i.e., not accounting for subdivision). Conclusively, we find that neglecting genetic substructure may lead to a massive underestimation of *N*_*e*_ and *N*_*b*_. Our findings should be investigated further using alternative methods, for example, with estimators based on sibship and parentage assignment (Jones & Wang, 2010; Wang et al., 2010). The results also suggest the need for further empirical analysis focusing on admixed individuals and their potential high influence on *N*_*b*_ and *N*_*e*_.

Results of ${\hat{N}}_{b_{\left(\mathrm{adj}\right)}}$ and ${\hat{N}}_{e_{\left(\mathrm{adj}\right)}}$ should be treated with caution, as the often assumed relationship *N*_*b*_ ≤ *N*_*e*_ **≤ generation length x *N*_*b*_ may not be reliable in many scenarios and is not eligible for iteroparous species with overlapping generations (Waples et al., 2013). In such species, a random sample of genotypes, which includes several generations, may underestimate true *N*_*b*_ (Waples et al., 2014). We pooled genotypes from individuals born over three years; hence, our results are not an exact estimation, but rather a related index of the true *N*_*b*_. Our goal was to trace estimates temporally with sufficient sample sizes (Hössjer, Olsson, Laikre, & Ryman, 2014), investigate the effects of population structure and admixture on the results, and test whether these changes track the reported demographic changes, as they may not shift concurrently (Bernos & Fraser, 2016; Yates, Bernos, & Fraser, 2017).

At present, our previous studies have shown that the genetic differentiation in the Finnish brown bear has gradually reached a low level (Hagen et al., 2015). Incorporating migration rates enables estimates of *N*_*e*_ based on asymmetric gene flow (Tufto & Hindar, 2003). However, the low population differentiation between the southern and northern cluster, especially in the later stages of population recovery in Finland, makes it challenging to estimate bidirectional migration rates. The degree of differentiation is below the threshold for a feasible estimation of gene flow or first‐generation migrants and may lead to biased or wrong results (Faubet, Waples, & Gaggiotti, 2007; Meirmans, 2014; Paetkau, Slade, Burden, & Estoup, 2004). Thus, in this system, migration rates would be more relevant for estimations of *N*_*e*_ on a larger geographical scale, including important source populations for the recovery, such as Russia. In such a scenario, a combination of empirical data and simulations may be used to estimate *N*_*e*_ under asymmetric gene flow (Palstra & Ruzzante, 2011; Pringle, Blakeslee, Byers, & Roman, 2011).

Increasing or maximizing *N*_*e*_ is often the goal of conservation efforts. However, in parts of Europe, where large carnivores such as the brown bear have recovered (Chapron et al., 2014), this goal has shifted toward keeping populations stable or even decreasing them slightly. In such cases, where reproduction is locally restricted to a few female‐core areas (Kojola, Danilov, Laitala, Belkin, & Yakimov, 2003; Swenson, Sandegren, & Söderberg, 1998) thus potentially leading to genetic substructure, the LD method may underestimate *N*_*e*_. Most wildlife management schemes operate on a national level and scientist are tasked to provide feasible results on a sound scale. Further, accounting for genetic subdivision and admixture may also sometimes be challenging, especially when subpopulations cannot be reliably identified. It may be tempting to relax the assumptions when estimating *N*_*b*_ and *N*_*e*_ in a natural system on national or international scale, where knowledge about population subdivision and/or migration may not be available. But, it has been shown that genetic drift and mixture had an effect on *N*_*b*_ based on LD in relation to the scale of the sampling area as a population living on a large geographical scale may consist of multiple, locally different genetic neighborhoods (Neel et al., 2013). The results of our study suggest that this should be carried out with caution and that tracing *N*_*b*_ and *N*_*e*_ in a natural and open system should account for population subdivision and admixture in order to reduce potentially severe upward or downward biases.

## CONFLICT OF INTEREST

None declared.

## DATA ARCHIVING STATEMENT

Data for this study is available at the Dryad depository under accession number: Provisional DOI: https://doi.org/10.5061/dryad.504g0; data files: Datafile_DRYAD.
