# Supplementary material for: Genetic substructure and admixture as important factors in linkage disequilibrium‐based estimation of effective number of breeders in recovering wildlife populations
Source: Ecol Evol. 2017 Nov 7;7(24):10721–32. doi: 10.1002/ece3.3577 (PMC5743533; doi:10.1002/ece3.3577)
Supplement: Supplementary file 1 [file ECE3-7-10721-s001.docx]

# Supplemental Material

Title:

**Genetic substructure and admixture as important factors in linkage disequilibrium based estimation of effective number of breeders in recovering wildlife populations**

Running title:

Number of breeders in recovering populations

**Table S1**: Raw results of the estimations of the effective number of breeders (raw$\hat{N}b$) for brown bears born in Finland 1993 to 2010 separated into six 3-year birth groups incl. estimations on the minimum census size (*Nc*) of brown bears, based on observations (see Material and Methods), sample sizes (*N*) and the raw estimates of effective number of breeders (raw$\hat{N}b$) from linkage disequilibrium based estimations with LDNE (Waples and Do, 2008) for the whole Finnish brown bear population, containing all data (raw$\hat{N}b$_FINLAND_), unambiguously assigned genotypes only (raw $\hat{N}b$_FINLAND_ _(ASSIGNED)_) as well as for the southern (raw$\hat{N}b$_SOUTH_) and northern (raw$\hat{N}b$_NORTH_) genetic cluster.

| Birth group | Minimum population size (*Nc*) | Sample sizes (*N*) | | | | Unadjusted estimates of effective number of breeders (raw $\hat{N}b$) | | | | | | | | | | | |
| --- | --- | --- | --- | --- | --- | --- | --- | --- | --- | --- | --- | --- | --- | --- | --- | --- | --- |
|  | *Nc*_MINIMUM_ | *N*_FINLAND_ | *N*_SOUTH_ | *N*_NORTH_ | *N*_ADMIXED_ | raw $\hat{N}b$_FINLAND_ | 95% CI | | raw $\hat{N}b$_FINLAND (ASSIGNED)_ | 95% CI | | raw $\hat{N}b$_SOUTH_ | 95% CI | | raw $\hat{N}b$_NORTH_ | 95% CI | |
| 1993-1995 | 686 | 79 | 49 | 25 | 5 | 161.9 | 123.9 | 226.8 | 151.2 | 113.2 | 219.6 | 90.7 | 45.6 | 686.5 | 207.4 | 129.9 | 467.4 |
| 1996-1998 | 783 | 158 | 80 | 60 | 18 | 130.8 | 113.1 | 153.1 | 119.5 | 101.5 | 143.1 | 59.3 | 47.8 | 75.9 | 166.2 | 127 | 233.9 |
| 1999-2001 | 845 | 116 | 61 | 43 | 12 | 141.9 | 118.5 | 174.1 | 130 | 104.4 | 168 | 75.1 | 54 | 116.4 | 187.8 | 129.4 | 325 |
| 2002-2004 | 815 | 127 | 63 | 45 | 19 | 165.6 | 136.7 | 206.5 | 143.7 | 116.2 | 184.3 | 150 | 91.9 | 355.1 | 208.3 | 140.9 | 376 |
| 2005-2007 | 840 | 96 | 46 | 40 | 10 | 197.6 | 151.3 | 277.1 | 160 | 113.6 | 254.5 | 108.2 | 67.8 | 233.9 | 180.2 | 114.1 | 389.6 |
| 2008-2010 | 1070 | 45 | 17 | 18 | 10 | 107.7 | 77.2 | 169.8 | 86.6 | 60.2 | 144.2 | 101.3 | 40.9 | ∞ | 87.8 | 41.6 | 8736.2 |
| Harmonic mean | 825.3 | 88.1 | 41.2 | 32.8 | 10.1 | 145.4 |  |  | 126.5 |  |  | 89.6 |  |  | 158.3 |  |  |
| SD | 126.9 | 39.4 | 21.2 | 15.0 | 5.3 | 31.2 |  |  | 26.5 |  |  | 31.3 |  |  | 44.7 |  |  |

**Table S2**: Average geographical latitude of the brown bears of the southern and northern genetic cluster as well as the average latitude of the admixed individuals of brown bears sampled in Finland for each 3-year birth group.

| Birth group | Latitude _SOUTH_ | Latitude _NORTH_ | Latitude _ADMIXED_ |
| --- | --- | --- | --- |
| 1993-1995 | 61.9254014 | 63.5145583 | 62.8994648 |
| 1996-1998 | 61.8358758 | 63.4273016 | 63.1668186 |
| 1999-2001 | 62.0156908 | 64.1858111 | 63.0450707 |
| 2002-2004 | 62.3377896 | 63.7611339 | 62.8074126 |
| 2005-2007 | 62.2679691 | 64.4224739 | 63.2916402 |
| 2008-2010 | 62.2522709 | 64.1879039 | 62.9414615 |

**
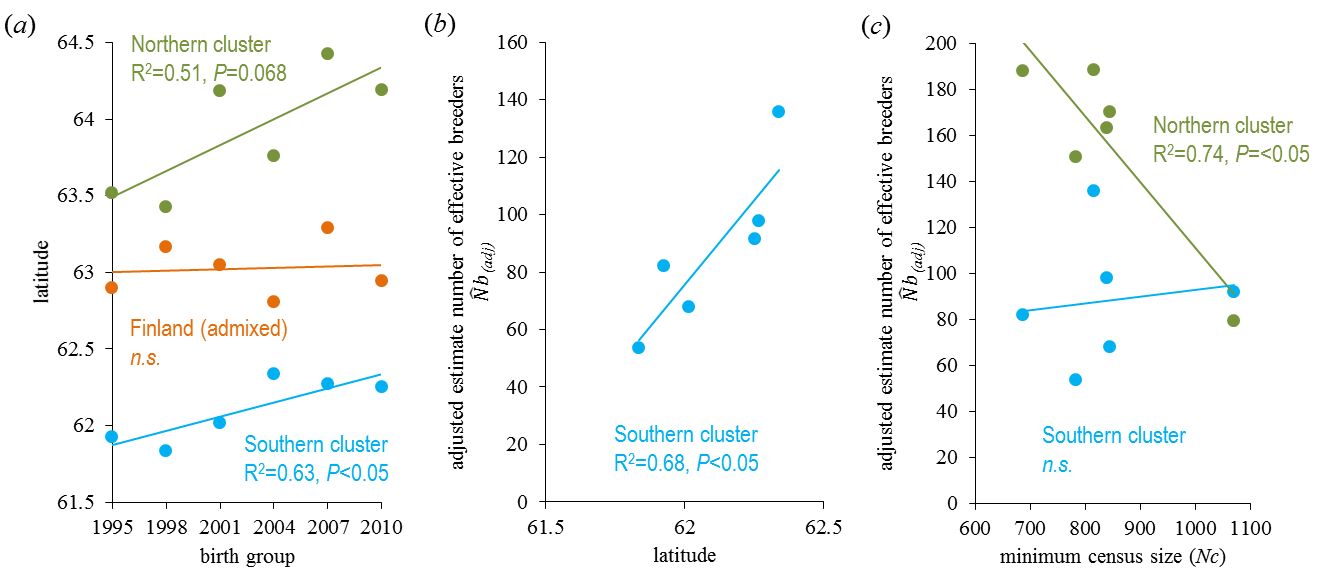
**

**Figure S1:** a) Range expansion of the Finnish brown bear population from 1993 to 2010 in birth groups combining 3-years and represented by the last year of the group on the x-axis. Temporal change of the mean latitude of the southern cluster (blue; b=0.003 (CI 0.003, 0.059), R^2^=0.63, *P*<0.05), northern cluster (green) cluster and unassigned, admixed genotypes (orange). b) Regression of the adjusted estimate of the number of effective breeders ($\hat{N}b$*_adj_*) of the southern cluster (blue) against the latitudinal shift of the southern cluster. c) Correlation of the adjusted number of effective breeders $\hat{N}b$*_adj_* in the identified southern (blue) and northern (green) genetic cluster versus the estimated minimum number of brown bears (*Nc*_MINIMUM_) in the country. See Table 3 for more statistical results.

**
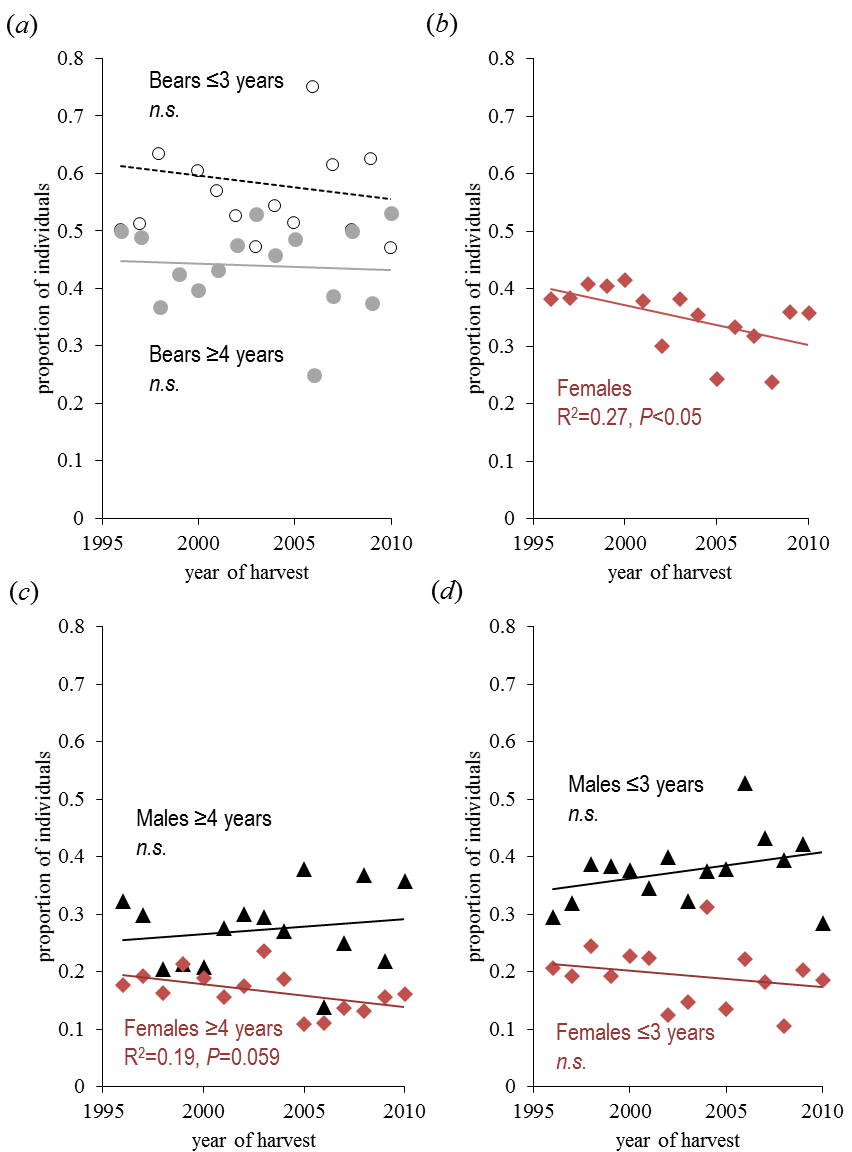
**

**Figure S2:** Proportion of brown bears in the analysed data from Finland, harvested 1996-2010: a) Proportion of brown bears of 1-3 years old (white circles) and brown bears older than 4 years (black circles). b) Proportion of females (red diamonds; b=-0.007 (CI -0.013, -0.001), R^2^=0.27, *P*<0.05) for each hunting year. c) Proportion of 4 years and older males (black triangles) and females (red diamonds). d) Proportion of 1-3 year old males (black triangles) and females (red diamonds).
